# Long-term growth outcomes in children with Hirschsprung disease after definitive surgery: A cross-sectional study

**DOI:** 10.1016/j.amsu.2020.09.038

**Published:** 2020-10-02

**Authors:** Gita Christy Gabriela, Esensi Tarian Geometri, Griselda Elisse Santoso, Kemala Athollah, Aditya Rifqi Fauzi, Janatin Hastuti, Gunadi

**Affiliations:** a Pediatric Surgery Division, Department of Surgery, Faculty of Medicine, Public Health and Nursing, Universitas Gadjah Mada/Dr. Sardjito Hospital, Yogyakarta, 55281, Indonesia; b Laboratory of Bioanthropology and Paleoanthropology, Faculty of Medicine, Public Health and Nursing, Universitas Gadjah Mada, Yogyakarta, 55281, Indonesia

**Keywords:** Hirschsprung disease, Long-term growth outcomes, Nutritional status, Transabdominal soave and duhamel surgeries, Transanal endorectal pull-through, HAEC, Hirschsprung-associated enterocolitis, HSCR, Hirschsprung disease, BAZ, body mass index-for-age z-score, ICD10, The International Statistical Classification of Diseases and Related Health Problems, 10th Revision, IQR, interquartile range, TEPT, transanal endorectal pull-through, WAZ, weight-for-age z-score, WHO, World Health Organization

## Abstract

**Background:**

The growth of children with Hirschsprung disease (HSCR) can be affected by many factors, including the environment, nutrient intake, and surgery. Our study compared the long-term (*i.e.,* at least 3 years of follow-up) growth outcomes in HSCR children after transabdominal Soave and Duhamel and transanal endorectal pull-through (TEPT) surgeries.

**Methods:**

A cross-sectional study was conducted in children <18 years of age diagnosed histopathologically with HSCR who underwent pull-through between January 1, 2012–December 31, 2015 in our institution. The postoperative anthropometric data were obtained prospectively through interviews during the outpatient clinic appointment or by telephone.

**Results:**

We recruited 21 patients (Soave: 7 *vs.* Duhamel: 4 *vs.* TEPT: 10; *p* = 0.06). There were no significant differences between the three surgical methods in terms of preoperative and postoperative nutritional status categories (*p* = 0.52). Concerning the changes in nutritional status, after Soave surgery, it was improved, steady, and worsened in 28.6%, 57.1%, and 14.3% of the children, respectively. The nutritional status of the Duhamel group was worsened and steady in 25% and 75% of the children, respectively, while in the TEPT group, it was improved and steady in 40% and 60% of the children, respectively. However, these differences were not statistically significant (*p* = 0.42).

**Conclusions:**

While some HSCR children show an improvement in their nutritional status after Soave and TEPT procedures, the overall nutritional status is similar among different procedures. Further multicenter studies with a larger sample size are important to clarify our findings.

## Highlights

• The growth of children with Hirschsprung disease (HSCR) can be affected by many factors, such as the environment, nutrient intake, and pull-through surgery.
• It is important to determine the long-term (*i.e.* at least 3 years of follow-up) growth outcomes in HSCR children after pull-through prospectively.
• While some HSCR children show an improvement in their nutritional status after Soave and TEPT procedures, the overall nutritional status is similar among different pull-through procedures.

## 1 Introduction

Hirschsprung's disease (HSCR) is a complex genetic disorder characterized by the absence of ganglion cells in the intestines [1,2]. One in 5000 births experiences this disorder [1,2], while in Indonesia, one in 3250 births has HSCR [3]. Seventeen genes have been identified to be involved in the development of HSCR [1,2]. The gold standard in diagnosing HSCR is full-thickness rectal biopsy [4,5]. Currently, most patients with HSCR are diagnosed in the newborn period [3,4]. Removing the aganglionic intestine through surgical resection has drastically reduced the mortality of this disease. Transabdominal Soave and Duhamel and transanal endorectal pull-through (TEPT) procedures are the most common surgeries performed for HSCR [[6], [7], [8]].

Since HSCR often occurs in newborns and young children, it is crucial to ensure that the quality of life, growth, and development of the child will not be disrupted. Their growth can be affected by many factors, such as the environment, nutrient intake, and surgery. Children with chronic diseases, such as HSCR, might develop growth and development disorders due to direct or indirect alteration of nutrient intake, parental interaction, or even from their social circle or peer group [9].

There are many parameters to assess children's growth. Body weight and height are the most universally used parameters. However, body weight is claimed to possess more sensitivity to the intake of food or nutrients compared to body height. Hence, weight fluctuates more easily than height. Linear growth insufficiency is usually caused by congenital, constitutional, familial, or endocrine factors instead of nutrient-related factors [10,11].

Our recent study determined the short-term growth outcomes in HSCR children after pull-through surgery [12]. Here, this study aimed to further compare the long-term (*i.e.* at least 3 years of follow-up) growth outcomes in children with HSCR after transabdominal and transanal pull-through procedures.

## 2 Material and methods

### 2.1 Patients

A cross-sectional study was conducted with the inclusion criteria of children <18 years of age diagnosed with HSCR [4] who underwent pull-through surgery between January 1, 2012–December 31, 2015 in Dr Sardjito Hospital, Indonesia, whereas the exclusion criteria were incomplete medical records and pull-through was not performed in our institution.

The diagnosis of HSCR in our institution was established using the clinical manifestations, contrast enema and histopathology as the gold standard [4].

The study was approved by the Institutional Review Board, Faculty of Medicine, Public Health and Nursing, Universitas Gadjah Mada/Dr. Sardjito Hospital (KE/FK/0855/EC/2017). Written informed consent was obtained from all parents for joining this study. Moreover, the study has been reported in line with the STROCSS criteria [13].

### 2.2 Pull-through

Transabdominal Soave, Duhamel and TEPT were performed according to previous studies [8,12,22] All transabdominal Soave and Duhamel procedures were conducted for patients with HSCR who had colostomy, whereas TEPT was performed for patients with HSCR without any prior colostomy.

### 2.3 Long-term growth outcomes

The postoperative anthropometric data were obtained prospectively through interviews during the outpatient clinic appointment or by telephone and assessed by the attending pediatric surgeons. The length of follow-up was at least 3 years after definitive pull-through surgery.

As anthropometric indices, weight-for-age z-score (WAZ) and body mass index-for-age z-score (BAZ) classifications from the World Health Organization (WHO) were used to evaluate the long-term growth outcomes. Anthropometric measurement of patients with HSCR included their weight and length measured preoperatively and at least ≥3 years [14] after the Soave, Duhamel, or TEPT surgery.

WAZ was classified into four categories as follows: a) severely underweight, b) underweight, c) normal, and d) overweight, whereas BAZ consists of five grades as follows: a) severely wasted, b) wasted, c) normal, d) overweight, and e) obese [15].

### 2.4 Statistical analysis

Data are presented as number/percentages and interquartile ranges (IQRs). The Fischer Exact was used to determine the differences between two independent proportions for nominal data, while the Kruskal-Wallis tests were used to evaluate the differences between groups for nonparametric values.

## 3 Results

### 3.1 Baseline characteristics

The International Statistical Classification of Diseases and Related Health Problems, 10th Revision (ICD-10) code of Q43.1 (HSCR) was used to identify patients diagnosed with HSCR. Eighty medical records were examined, and 59 were excluded due to incomplete medical records, deceased patients, or refusal to be contacted; thus, we further analyzed 21 patients. Twenty-one patients with HSCR (Soave 7 *vs*. Duhamel 4 *vs.* TEPT 10; *p* = 0.06) had complete data for the final analysis. None of the clinical characteristics of the patients with HSCR showed any significant difference between the three surgical methods (Table 1). All patients had nonsyndromic HSCR, without any neurodevelopmental delay or comorbidity.

**Table 1:** Baseline characteristics of HSCR patients who underwent transabdominal Soave, Duhamel and TEPT procedures in our institution.

Characteristic	Soave (n, %; median, IQR)	Duhamel (n, %; median, IQR)	TEPT (n, %; median, IQR)	*p*-value
Sex				0.06
Male	7 (100)	3 (75)	5 (50)	
Female	0	1 (25)	5 (50)	
Age at definitive surgery (years)	1.8 (1.25–3.8)	2.15 (1.13–3.25)	1.65 (0.18–3.48)	0.33
Age at interview (years)	6.4 (4.9–8.55)	8.35 (7.38–8.43)	5.35 (3.65–7.78)	0.33
Length of follow-up (years)	4.6 (3.5–5.1)	4.8 (3.95–6.08)	3.58 (3.07–4.48)	0.32
Preoperative body weight (kg)	10 (8.95–11)	9.9 (7.53–12.25)	8.1 (4.43–12.13)	0.64
Postoperative body weight (kg)	17 (15.25–18)	17.5 (14.25–21.5)	16.25 (13.43–22.65)	0.77
Preoperative height (cm)	104 (98.5–116)	74^a^	77 (47.75–106.75)	–
Postoperative height (cm)	105 (100–114.5)	107.5 (96.25–118.75)	101 (97.75–118)	0.81
Postoperative complication				
Constipation	3 (42.9)	1 (25)	0	0.32
Soiling	2 (28.6)	3 (75)	8 (80)	0.85
HAEC	1 (14.3)	0	0	–
None	2 (28.6)	1 (25)	2 (20)	0.92
Employment status				0.31
Employed	3 (42.9)	0	3 (30)	
Not-employed	4 (57.1)	4 (100)	7 (70)	

a Only one medical record noted the body height in the Duhamel group; HAEC, Hirschsprung-associated enterocolitis; HSCR, Hirschsprung disease; IQR, interquartile range; TEPT, transanal endorectal pull-through.

### 3.2 Comparison of nutritional status of HSCR patients after different pull-through procedures

Next, we compared the preoperative and postoperative nutritional status according to the WHO WAZ categories between the three different procedures. There were no significant differences between the three surgical methods in terms of preoperative and postoperative nutritional status categories (*p* = 0.52) (Table 2).

**Table 2:** Comparison of preoperative and postoperative nutritional status categories of patients with HSCR following Soave, Duhamel and TEPT procedures according to the WHO WAZ.

Nutritional Status	TEPT		Duhamel		Soave		*p*-value
	Preoperative (n, %)	Postoperative (n, %)	Preoperative (n, %)	Postoperative (n, %)	Preoperative (n, %)	Postoperative (n, %)	
Severely underweight	2 (20)	0	0	1 (25)	2 (28.6)	0	0.52
Underweight	1 (10)	2 (20	2 (50)	2 (50)	1 (14.3)	3 (42.9)**	
Normal	6 (60)	8 (80)	2 (50)	1 (25)	4 (57.1)*	4 (57.1)**	
Overweight	1 (10)	0	0	0	0	0	

* and **: One patient (normal) pre- and two patients (normal and underweight) post-Soave, showed an age of >10 years old, and their nutritional status was calculated using the WHO BAZ; TEPT, transanal endorectal pull-through.

Subsequently, we classified the changes in nutritional status after pull-through into three categories: improved, steady and worsened. The nutritional status after Soave surgery was improved, steady, and worsened in 28.6%, 57.1%, and 14.3% of the children, respectively. In the Duhamel group, it was worsened and steady in 25% and 75% of the children, respectively, while in the TEPT group, it was improved and steady in 40% and 60% of the children, respectively. However, these differences were not statistically significant (*p* = 0.42) (Table 3).

**Table 3:** Changes in the nutritional status of HSCR patients following Soave, Duhamel and TEPT procedures in our institution.

Nutritional status changes	Soave (n, %)*	Duhamel (n, %)	TEPT (n, %)	*p*-value
Improved	2 (28.6)	0	4 (40)	0.42
Steady	4 (57.1)	3 (75)	6 (60)	
Worsened	1 (14.3)	1 (25)	0	

*, For two patients after the Soave procedure, their nutritional status was calculated using BAZ and showed improved nutritional status; TEPT, transanal endorectal pull-through.

## 4 Discussion

In this study, we present the changes in nutritional status in patients with HSCR after three different procedures with a length of follow-up of at least three years. None of the pull-through procedures had better growth outcomes (*i.e.,* improvement of nutritional status) than the others, although ~30% and 40% of patients with HSCR showed improved nutritional status after Soave and TEPT, respectively (Table 3). These findings further confirmed the results of previous studies [12,16]. At least four novelties were noted in our study: 1) we compared growth outcomes between three different pull-through surgeries (*vs.* two different procedures [16]); 2) longer period of follow-up (≥3 years) (*vs.* short-term follow-up [12,16]); 3) prospective design for postoperative anthropometric data (*vs.* retrospective study [12,16]); and 4) we used WAZ and BAZ indices to determine the growth outcomes (*vs.* weight and length at one year of age [16]).

We determined the length of follow-up of at least ≥3 years according to a previous study [14]. The average length of follow-up in our study was approximately 4–5 years after surgery (Table 1), while our previous report did not determine the minimum length of follow-up with the longest period of follow-up of two years after pull-through [12]. Three years of follow-up is considered sufficient to represent the time needed to see the long-term growth of patients with HSCR after pull-through procedures [14].

Several variables might affect our results. First, age differences may affect the results of the measurements of patients' body weight and height both before and after surgery periods, especially since in childhood, the growth of children occurs rapidly in the early period, then slows, and again becomes rapid during growth spurt periods, usually at 10 years of age for females and 12 years of age for males [17]. Therefore, the greater the difference in age of subjects at the time of the measurements, the greater it may affect the results of the study. Three patients in our study were >10 years old, including one child in preoperatively and two children in postoperatively from the Soave group (Table 2). Second, BMI may differ between populations, which might reflect different levels of physical activity and provision of nutrition for children in different populations [18]. Third, different diets contain a variety of nutrients. For example, there will be differences between a breastfed child and a non-breastfed child over time. A child who was not breastfed will have a higher chance of becoming overweight [19]. Unfortunately, our study did not include the patients' nutrition as a variable, becoming a weakness of our report. Instead, we used the parents' employment status to represent their socioeconomic status which might affect the nutritional status of their children. The parents' employment status was not significantly different between groups (Table 1). The association between parents' employment status and children's nutritional status is still controversial [[20], [21], [22]]. Some other factors might also affect the nutritional status of children, such as the child's food intake, the parents' educational status and knowledge regarding the importance of balanced nutrition and exercise, and the role of family or persons who take care of children when both parents work [21,22].

Additionally, postoperative complications might affect the growth outcomes of children with HSCR. A previous study showed that the frequency of constipation was higher in patients after the Soave procedure than after the Duhamel procedure, while the soiling rate was similar in both groups [23]. Moreover, the TEPT group showed less soiling and constipation than those who underwent transabdominal pull-through procedures [24]. Our study revealed that complications after surgery were not significantly different among the three different procedures (Table 1).

Notably, the small number of participants is a limitation of our study. These small sample sizes might result in a lack of study power and significance. One of the reasons for the small sample size was that most parents refused to participate because their children are already in good health, and the study does not have any direct impact on their children's outcomes. In addition, the pull-through methods, whether the transanal or transabdominal approach was chosen according to the attending physician preference and the patients' condition, such as age at HSCR diagnosis. We are also unable to control other variables that might affect children's growth, such as environment and nutrient intake, since we did not determine the nutritional assessment of the children with trace elements or other serology. Moreover, prior colostomy in the Soave and Duhamel groups and the presence of Hirschsprung-associated enterocolitis (HAEC) after pull-through might affect the patients' nutritional status. Another limitation of our study is its retrospective design; therefore, some data might be unrecorded. These facts should be considered during the interpretation of our findings.

In conclusion, while some HSCR children show an improvement in their nutritional status after Soave and TEPT procedures, the overall nutritional status is similar among different pull-through procedures. Further multicenter studies with a larger sample size are important to clarify our findings.

## Ethical approval

The study was approved by the Institutional Review Board, Faculty of Medicine, Public Health and Nursing, Universitas Gadjah Mada/Dr. Sardjito Hospital (KE/FK/0855/EC/2017).

## Sources of funding

This study was funded by Indonesian Ministry of Research and Technology/National Agency for Research and Innovation.

## Author contribution

Gunadi, Gita Christy Gabriela, Esensi Tarian Geometri, and Griselda Elisse Santoso conceived the study. Gunadi, Aditya Rifqi Fauzi and Kemala Athollah drafted the manuscript. Janatin Hastuti critically revised the manuscript for important intellectual content. All authors read and approved the final draft. All authors facilitated all project-related tasks.

## Registration of research studies

Researchregistry5218.

## Guarantor

Gunadi.

## Consent

Written informed consent was obtained from the parents’ patients for publication of this article. A copy of the written consent is available for review by the Editor-in-Chief of this journal on request.

## Provenance and peer review

Not commissioned, externally peer reviewed.

## Declaration of competing interest

No potential conflict of interest relevant to this article was reported.
